# Progressions in Cardiac Arrhythmia: Specific Populations and the Need for Precision Medicine

**DOI:** 10.3390/jpm13071122

**Published:** 2023-07-11

**Authors:** José Miguel Rivera-Caravaca, Jeroen M. Hendriks

**Affiliations:** 1 Faculty of Nursing, University of Murcia, 30120 Murcia, Spain; 2 Department of Cardiology, Hospital Clínico Universitario Virgen de la Arrixaca, Instituto Murciano de Investigación Biosanitaria (IMIB-Arrixaca), Centro de Investigación Biomédica en Red—Enfermedades Cardiovasculares (CIBERCV), 30120 Murcia, Spain; 3 Caring Futures Institute, College of Nursing and Health Sciences, Flinders University, Sturt Road, Bedford Park South Australia 5042, GPO Box 2100, Adelaide, SA 5001, Australia; jeroen.hendriks@flinders.edu.au; 4 Centre for Heart Rhythm Disorders, University of Adelaide and Royal Adelaide Hospital, Port Road, Adelaide, SA 5001, Australia

Atrial fibrillation (AF) is the most common cardiac arrhythmia in the general population. The high prevalence of this condition—which is expected to be even higher in upcoming years [1]—has motivated in-depth studies, from mechanistic and pathophysiological approaches to further understand how AF is initiated and perpetuated to analyses of the role and impact of integrated care in its management. In this Special Issue of the *Journal of Personalized Medicine*, some controversial and scarcely investigated topics related to AF have been introduced, with the aim of providing holistic care and treatment in AF. These topics include improving the detection, adverse outcome prevention, and risk factor optimization in AF patients.

Escribano et al. aimed to conduct an analysis of the f-wave harmonic spectral structure to improve catheter ablation (CA) outcome prediction through several entropy-based measures computed on different frequency bands in 151 patients with persistent AF under radio frequency CA. Remarkably, the authors demonstrated in this pioneering analysis of the f-wave harmonic spectral structure that the presence of larger harmonics and a proportionally smaller dominant frequency peak was strongly associated with a decreased probability of AF recurrence after CA [2].

To better understand the CA process, Vraka and colleagues have decomposed crucial CA steps, including coronary sinus catheterization and the impact of left and right pulmonary vein isolation (PVI). In brief, they showed that left PVI is the critical part of the CA of PVs for paroxysmal AF patients, significantly altering the P-wave duration, whereas the effect of the CA of PVs on the coronary sinus is less straightforward and is demonstrated to a lesser extent. The authors suggest that other atrial structures may be more indicative of the ablation outcome and should be assessed as alternative references [3].

Osorio et al. compared the performance of cycle length estimations of three different local activation wave detection methods: the hyperbolic tangent (HT) function, an adaptive amplitude threshold (AAT), and a cycle length iteration (ACLI). For the HT method, the accuracy, sensitivity, and precision were higher compared to the AAT and ACLI methods, with even a lower cycle length error. The authors concluded that the high robustness and precision demonstrated by the HT method promote its implementation on CA mapping devices for a more successful location of ablation targets and improving the results of CA procedures [4].

Currently, left atrial (LA) appendage surgical exclusion or percutaneous occlusion have limitations. For this reason, an interesting study by Pasta et al. sought to quantify the hemodynamic and structural loads of a novel potential procedure to partially invert the “dead” LA appendage space to eliminate the auricle apex, called LA appendage inversion (LAAI). This procedure was simulated by pulling the elements at the LA appendage tip and prescribing a displacement motion along a predefined path. Later, the authors used the deformed configuration to develop a computational flow analysis of LAAI, demonstrating that the inverted LAA wall in the inverted appendage undergoes a change in the stress distribution from tensile to compressive, and this can lead to resorption of the LAA tissue as per a reduced stress/resorption relationship [5].

From a clinical point of view, three original studies performed in-depth analyses of the management of AF. Merino-Merino and collaborators, in a confirmatory study including patients with AF and healthy controls, showed that NT-proBNP, high sensitivity troponin T, and ST2, were significantly related to the presence of AF. Among these biomarkers of cardiac dysfunction, inflammation, and damage, NT-proBNP exhibited the best discrimination ability (with an area under the curve of 0.995) [6]. Pastori et al. identified clinical phenotypes of AF patients to stratify the mortality risk by a cluster analysis performed on 5171 AF patients from the nationwide START (survey on anticoagulated patients register). In brief, cluster 3, characterized by men with diabetes, coronary disease, and peripheral artery disease, and cluster 4, including mainly elderly women with previous cerebrovascular events, had a higher risk of mortality (aHR 6.702, 95% CI 2.433–18.461, and aHR 8.927, 95% CI 3.238–24.605; respectively) [7]. Roldán et al. presented the first study analyzing the impact of adherence to the Atrial Fibrillation Better Care (ABC) pathway on the quality of anticoagulation control in a cohort of AF outpatients starting vitamin K antagonists. Amongst ABC pathway non-adherent patients, a greater proportion had TTR < 65% or TTR < 70%, with a lower mean TTR in non-adherent patients (59.4 ± 22.3% vs. 63.9 ± 21.1%; *p* = 0.004). Indeed, adherence to the ABC pathway was independently associated with a TTR of ≥65% [8].

Additionally, two reviews aimed to shed light on very specific patient populations and therapeutic interventions. In the first review, Badescu and colleagues summarized the current evidence concerning the management of AF in people with hemophilia, which is a complex combination in terms of the prevention of bleeding and thromboembolic complications. These patients are often perceived as having a high bleeding risk due to coagulation factor VIII/IX deficiency, but in the presence of AF they also have a high risk of stroke and systemic embolism. Despite the lack of research in this population, it should be highlighted that the treatment offered to the general population, including CA and LA appendage occlusion, can be implemented in hemophiliacs if an appropriate replacement therapy can be provided [9]. Buckley et al. reviewed, synthesized, and proposed an AF-specific exercise rehabilitation guideline based on data from primary trials and real-world cohort studies. According to their analysis, a minimum exercise threshold of 360–720 metabolic equivalent minutes/week, corresponding to 60–120 min of exercise per week at moderate-to-vigorous intensity, could be an evidence-based recommendation for patients with AF. This would improve AF-specific outcomes, quality of life, and possibly prevent long-term major adverse cardiovascular events. Non-traditional, low–moderate intensity exercise, such as yoga, appears to have promising benefits to patient quality of life and should therefore be considered in a personalized rehabilitation program [10].

Finally, AF is generally underdiagnosed, mainly because it can be present without signs and symptoms. For this reason, the real prevalence of AF is assumed to be higher than the known prevalence. In this Special Issue, Jones et al. described the protocol of an interesting study with the objective of investigating the effectiveness of a hand-held device embedded into the handles of supermarket trolleys in screening for AF in the general population. This will be a mixed method two-phase study. The quantitative first phase will be a cross-sectional observational study involving participants from a convenience sample at four large supermarkets with pharmacies. The prospective participants will be asked to conduct their shopping using a trolley embedded with a MyDiagnostick single-lead electrocardiogram sensor. If the device identifies a participant with AF, the in-store pharmacist will be dispatched to take a manual pulse measurement and a static control sensor reading and offer a cardiologist consultation referral. In the qualitative second phase, semi-structured interviews carried out with those pharmacists and store managers during the running of the trial period will be performed to explore the perceptions of staff regarding the benefits of the device [11].

In summary, the studies included in this Special Issue cover different areas in the field of AF and provide an updated overview of the complexities of AF management. These novel insights are of great interest to healthcare professionals treating patients with AF and may assist in decision-making processes.
